# Brain Areas Critical for Picture Naming: A Systematic Review and Meta-Analysis of Lesion-Symptom Mapping Studies

**DOI:** 10.1162/nol_a_00097

**Published:** 2023-04-11

**Authors:** Vitória Piai, Dilys Eikelboom

**Affiliations:** Radboud University, Donders Centre for Cognition, Nijmegen, Netherlands; Radboudumc, Donders Centre for Medical Neuroscience, Department of Medical Psychology, Nijmegen, Netherlands; Max Planck Institute for Psycholinguistics, Nijmegen, Netherlands

**Keywords:** confrontation naming, lexical semantics, object naming, oral naming, word finding

## Abstract

Lesion-symptom mapping (LSM) studies have revealed brain areas critical for naming, typically finding significant associations between damage to left temporal, inferior parietal, and inferior fontal regions and impoverished naming performance. However, specific subregions found in the available literature vary. Hence, the aim of this study was to perform a systematic review and meta-analysis of published lesion-based findings, obtained from studies with unique cohorts investigating brain areas critical for accuracy in naming in stroke patients at least 1 month post-onset. An anatomic likelihood estimation (ALE) meta-analysis of these LSM studies was performed. Ten papers entered the ALE meta-analysis, with similar lesion coverage over left temporal and left inferior frontal areas. This small number is a major limitation of the present study. Clusters were found in left anterior temporal lobe, posterior temporal lobe extending into inferior parietal areas, in line with the arcuate fasciculus, and in pre- and postcentral gyri and middle frontal gyrus. No clusters were found in left inferior frontal gyrus. These results were further substantiated by examining five naming studies that investigated performance beyond global accuracy, corroborating the ALE meta-analysis results. The present review and meta-analysis highlight the involvement of left temporal and inferior parietal cortices in naming, and of mid to posterior portions of the temporal lobe in particular in conceptual-lexical retrieval for speaking.

## INTRODUCTION

According to psycholinguistic models of language production, a speaker starts with a concept they want to express and goes through several stages until their intention can be articulated. Generally speaking, these stages can be seen as conceptual preparation, lexical selection (i.e., an operation at the level of “lemmas,” a semantic-syntactic representation), phonological retrieval and encoding (i.e., the retrieval and ordering of the speech sounds associated with that lemma), phonetic encoding (i.e., the computation of the gestural score), and articulation (e.g., Dell, 1986; Dell & O’Seaghdha, 1992; Levelt et al., 1999).

Producing language involves an extensive network of brain areas. Neurolinguistic models of language production have attempted to link the proposed cognitive stages to different brain areas (Hickok & Poeppel, 2007; Indefrey & Levelt, 2004; Roelofs, 2014). Various methods have been used to uncover these neural substrates, for example, by combining word production tasks with functional neuroimaging (e.g., Price et al., 1996), electrophysiology (e.g., Liljeström et al., 2009), or neurostimulation techniques (e.g., Hernandez-Pavon et al., 2014). In general, these methods have highlighted the correlation between activity in a broad fronto-temporo-parietal network and word production.

A different, noncorrelational approach to studying language production consists of examining the consequences of tissue damage on performance, as done with lesion-symptom mapping (LSM) techniques. Although differing in the particular methodology, these techniques aim to map the relationship between lesions and behavior, generating statistical maps. Rather than requiring a cut-off score or binary data (e.g., behavioral performance is either deficient or not), a continuous behavioral score can be used, thus enabling a more sensitive approach. Furthermore, these techniques can serve as a whole-brain analysis, rather than studying particular regions-of-interest (ROIs), although they are inherently limited by lesions coverage. In one particular and much used approach, voxel-based lesion-symptom mapping (VLSM), a statistical test is run at every voxel, comparing a behavioral score between patients with and without a lesion in that voxel, thus identifying voxels critical for the measured behavioral performance (Bates et al., 2003). Besides VLSM, similar (and more recent) lesion-symptom mapping approaches exist, such as voxel-based morphometry (Ashburner & Friston, 2000), voxel-based correlational methodology (VBCM; Tyler et al., 2005), and multivariate methods (reviewed in Ivanova et al., 2021), such as support vector regression multivariate LSM (Zhang et al., 2014). We note that a discussion of how these methods work is beyond the scope of the present study (see for explanation and comparisons between these methods, e.g., Geva et al., 2012; Ivanova et al., 2021).

Baldo et al. (2013) applied VLSM on naming accuracy in chronic stroke patients, while controlling for overall fluency in speech production and visual recognition of the items in the naming test. Significant brain regions were predominantly found in the left mid and posterior portions of the middle temporal gyrus (MTG), suggesting that naming critically depends on this area and the adjacent white matter. A different study by Thye and Mirman (2018) also found that damage to the left MTG was associated with deficits in naming in stroke patients, in addition to areas in the left inferior frontal gyrus (IFG), supramarginal gyrus (SMG), and angular gyrus (AG). Similar LSM studies confirmed the involvement of these areas but also found other or additional areas associated with naming performance, such as the left postcentral gyrus, inferior temporal gyrus (ITG), inferior longitudinal fasciculus, and temporal pole (e.g., Alyahya et al., 2018b; Faroqi-Shah et al., 2014; Piras & Marangolo, 2007).

### Present Study

In sum, damage to left temporal, inferior parietal (AG and SMG) and inferior frontal areas is typically associated with deficits in naming, though specific subregions found in the literature vary. An informal attempt to summarize available LSM evidence may be complicated by the fact that comparability is limited when different studies implement different experimental designs. Studies may for example vary in employed LSM approach, task demand, and covariates used. Furthermore, small sample sizes may be investigated, resulting in lower reliability. However, the use of a formal meta-analytic approach allows for a quantitative review of a large body of LSM data, enabling the identification of locations in the brain that show consistent relationships to behavior across studies (Eickhoff et al., 2012). Hence, the aim of the present study was to perform a systematic review and an anatomic likelihood estimation (ALE) meta-analysis of studies using LSM methods in combination with a naming task, to identify a pattern of consistent associations between brain lesions and word production. In addition, a more in-depth analysis was performed attempting to align the processing stages most likely tapped into by a meta-analyzed study to stages proposed by psycholinguistic models of word production (e.g., Dell, 1986; Dell & O’Seaghdha, 1992; Indefrey & Levelt, 2004; Levelt et al., 1999).

**Figure 1.**
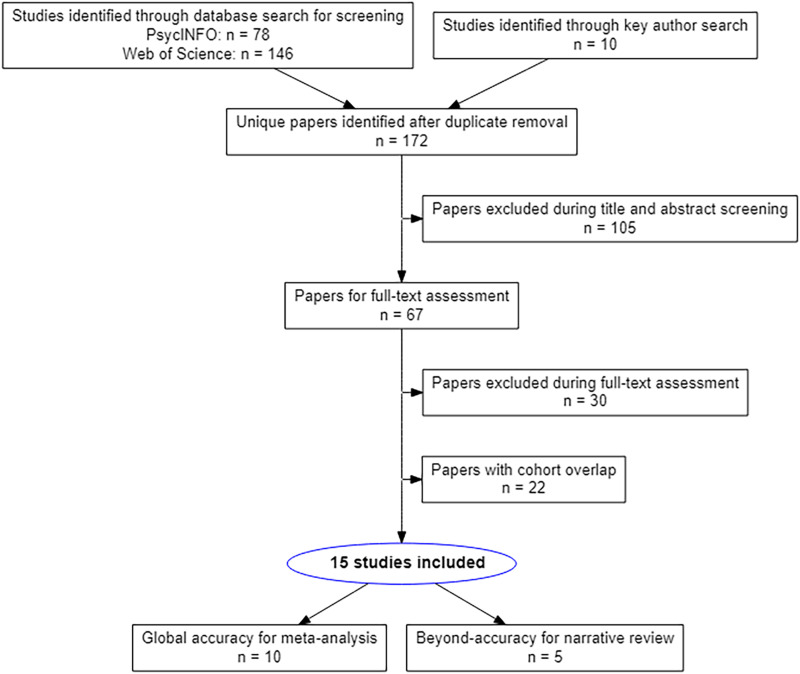
PRISMA flow diagram of the literature selection process.

In our systematic review, papers using any form of LSM were considered. Papers had to implement oral naming as a behavioral task in some form. To limit heterogeneity across the researched participant group, we only included studies in individuals beyond the acute stages of stroke (here defined as at least one month post-stroke). Papers in which the dependent variable was global accuracy in naming performance were considered for global accuracy analysis. From these, only papers that provided coordinates qualified for ALE meta-analysis (see Figure 1). Papers with a dependent variable more elaborate than global accuracy, such as error type in naming or combining naming with another language task, were not considered for the ALE meta-analysis since there was not enough consistency across them, which would introduce large heterogeneity in the dependent variable tested. Instead, these papers were considered for a beyond-accuracy analysis in narrative form. Dependent variables from these papers were linked to the stages of word production described above in an attempt to elucidate the ALE meta-analysis results.

## MATERIALS AND METHODS

### Literature Search and Selection

A systematic search was performed, using the Web of Science Core Collection (Clarivate, 2023) and APA PsycINFO (American Psychological Association, 2023) databases using the following keywords: [("naming" OR "production") AND ("stroke" OR "poststroke" OR "infarct" OR "CVA" OR "cerebral vascular accident*" OR "cerebrovascular accident*" OR "post stroke") AND ("lesion behavio*r mapping" OR "voxel wise" OR "voxel based" OR "symptom mapping" OR "lesion mapping")]. This resulted in a list of 162 unique papers (last updated on September 18, 2021). To identify other papers not picked up in the automatic search, publications from the following authors (who have published seminal studies with LSM methodology) were screened: Baldo, J.; Binder, J. R.; Crinion, J. T.; Dragoy, O.; Fridriksson, J.; Hope, T. M. H.; Lambon Ralph, M. A.; Mirman, D.; Price, C. J.; Schwartz, M. E.; and Wilson, S. M. Furthermore, the reference list of a review paper by Mirman and Thye (2018) was searched for relevant papers. This yielded 10 additional papers. A PRISMA flowchart showing the selection of these papers can be found in Figure 1.

Papers were independently screened by two authors (title and abstract in phase one, full text in phase two) on whether they satisfied the following selection criteria: papers had to (1) be original empirical work; (2) include stroke patients only; (3) state clearly that all patients were at least one month post onset of stroke; (4) involve LSM of (5) oral picture naming task performance; (6) not reporting single cases; (7) not based on functional imaging or using synthetic data; (8) not studying predefined ROIs. During full-text screening, papers (with corresponding total number) were excluded according to the following criteria: Not single-word oral picture naming (*N* = 13); no LSM methodology (*N* = 6); not all patients >1 month post-stroke (*N* = 4); not (only) stroke cohort (*N* = 2); no coordinates provided (*N* = 3); no original or real data (*N* = 2).

### Paper Categorization

Papers were categorized according to the dependent variable used for LSM, resulting in a global accuracy analysis data set (i.e., accuracy in naming performance, not further specified). An exception was made for papers studying a compound naming score from the Western Aphasia Battery (Kertesz, 1982). This score consists of four naming subtests, one of which is oral naming, which we considered to suffice for our analyses, thus allowing us to include more papers in the ALE meta-analysis. Papers in the global accuracy analysis data set had to provide coordinates to be included for ALE meta-analysis. Authors were contacted for additional information on foci coordinates. Papers analyzing a more specific score, for example, the number of semantic or phonological errors or after a dimensionality reduction step (e.g., using principal component analysis [PCA]), were included for a beyond-accuracy analysis.

Next, for each type of analysis (i.e., global accuracy or beyond-accuracy), relevant papers were screened for potential overlap in the participant sample by checking overlap in authors and noting where participants were recruited from. For unclear cases, authors were contacted to gain information regarding the participants tested, but no response was received. To reduce the risk of duplicated data, which inflates the effect size, from every subgroup of potentially overlapping papers, the paper that suited our research purpose best was selected in the following way. If dependent variables used in the overlapping papers were equal, the paper with the largest cohort was selected. Priority was given to papers providing coordinates over papers without them. Selection priority was also given to the dependent variable reflecting the most clear-cut measure of naming (as opposed to the naming score being used with a technique for dimension reduction such as PCA, which is then related to lesion information), noun naming specifically rather than verb naming (since verb naming was much less common across the studies). If multiple LSM analyses were performed within one paper, selection priority was given to results from analyses of which noun naming was the largest part, as this is the most commonly reported measure of naming across papers. Also, univariate VLSM results were prioritized over any other form of LSM, and so were manually rather than automatically traced lesion maps, as these two types tend to be the most common approaches. Furthermore, priority was given to results controlling for all available covariates. The choice for the most commonly used approach was meant to increase the comparability across studies and, thus, decrease heterogeneity.

For the ALE meta-analysis, clusters of (potentially) overlapping papers were found. Out of the first cluster (Alyahya et al., 2018a, 2018b; Butler et al., 2014), the paper by Alyahya et al. (2018b) was selected for including noun naming (cf. Alyahya et al., 2018a) and a more suitable dependent variable to our study (cf. Butler et al., 2014). The second cluster consisted of two papers (Piras & Marangolo, 2007, 2010), for which Piras and Marangolo (2010) provided coordinates. For the third cluster (Pustina et al., 2016; Thye & Mirman, 2018; Zhang et al., 2014), the paper by Thye and Mirman (2018) was selected given the availability of foci coordinates. For the two papers by Lukic et al. (2017, 2021), using verb naming, the one with the larger sample size was chosen (Lukic et al., 2021).

For beyond-accuracy analysis, clusters of (potentially) overlapping papers were obtained. The first cluster contained three papers (Fridriksson et al., 2016, 2018; Stark et al., 2019), out of which Fridriksson et al. (2018) was selected for studying different error types within naming (cf. Fridriksson et al., 2016) and the larger sample size (cf. Stark et al., 2019). The second cluster consisted of nine papers (Alyahya et al., 2018a, 2020a, 2020b; Butler et al., 2014; Halai et al., 2017, 2018a, 2018b; Tochadse et al., 2018; Zhao et al., 2020), out of which Tochadse et al. (2018) was selected. In this paper, semantic and phonological errors were studied, yielding *s* and *p* parameters, respectively, according to the computational model of Dell (Dell, 1986; Dell et al., 2013), whereas all other papers in this cluster used PCA on a neuropsychological test battery, except for Halai et al. (2018b). This latter paper studied different error types within naming but was excluded as Tochadse et al. (2018) provided, in our opinion, a theoretically better motivated distinction between error types than Halai et al. (2018b). The final cluster consisted of nine papers (Chen et al., 2019; Dell et al., 2013; Mirman, Chen, et al., 2015; Mirman & Graziano, 2013; Mirman, Zhang, et al., 2015; Schwartz et al., 2009, 2011, 2012; Walker et al., 2011), of which Dell et al. (2013) was chosen for conducting VLSM on parameters derived from computational modeling (as in Tochadse et al., 2018).

The selection yielded 15 original research papers that used LSM techniques in combination with a (picture) naming task in stroke individuals >1 month post-onset with non-overlapping cohorts. Ten papers qualified for ALE meta-analysis of global accuracy and five for beyond-accuracy analysis. The selection and categorization procedure of the included papers can be found in a PRISMA flowchart, shown in Figure 1.

### Quality Assessment

To try to chart the heterogeneity across studies included in the meta-analysis, we performed a quality assessment of the evidence for the purpose of our systematic review by checking various parameters. We note that this does not speak to the quality of the papers themselves, but rather to the quality of the evidence as it impacts our synthesis and findings. Papers could receive a maximum of 6 points on the (clarity of the description of the) studied population, 2 points for the clarity of the description of the task and how performance was scored, 4 points for the (description of the) statistical analysis, and finally 1 point for the clarity of the outcome measure, with a maximum of 13 points in total. Details regarding the parameters and weighted distributions of points can be found in Table S1 in the Supporting Information, available at https://doi.org/10.1162/nol_a_00097, and scoring per paper can be found in Table S2.

### ALE Meta-analysis

ALE meta-analysis was performed using the revised version implemented in BrainMap GingerALE 3.0 software following the MNI152 template (Eickhoff et al., 2009, 2012; Turkeltaub et al., 2012), in conjunction with anatomical data (rather than using functional neuroimaging data for activation likelihood estimation meta-analyses, see for a similar approach Na et al., 2022; Urgesi et al., 2014). These data concerned peak coordinates of significant clusters associated with naming task performance as a result of LSM. All extracted coordinates were reported in Montreal Neurological Institute (MNI) space.

In ALE analysis, foci from a given study or experiment are modeled as Gaussian probability density distributions with a full-width half-maximum (FWHM) calculated from the experiment’s sample size and merged together to form a map. This map therefore represents a summary of the results of that study, taking into account between-subject and between-template variability (e.g., caused by data smoothing and standardization into anatomical space), by modeling foci as probability distributions rather than singular points. These probability distributions are then taken together by calculating the voxel-wise union of the maps from different studies, to assign to every voxel an ALE value equal to the probability that at least one of the foci in the data set actually lies within this voxel (Turkeltaub et al., 2002, 2012). Lastly, convergence of foci across experiments is tested by comparing the calculated ALE values against ALE values obtained under an empirically defined null distribution reflecting random spatial association. A whole-brain map can then be produced, showing the differential likelihood of associations (in our case, between lesion and naming score) at all brain locations afforded by the lesion coverage. Significance was assessed using a cluster-level familywise error correction set at *p* < 0.05, with a cluster forming threshold set at *p* < 0.01 and 1000 permutations. Anatomical labels were obtained from BrainMap GingerALE 3.0, based on the Talairach Daemon (1988 Talairach atlas). We note that the use of a Gaussian probability density distribution with FWHM, which is commonly used for functional magnetic resonance imaging (fMRI) studies, may not be the best option for an ALE meta-analysis of LSM studies. However, fMRI is a haemodynamic measure shaped by properties of the vascular system and strokes are vascular in nature, motivating the use of this distribution. This issue remains nevertheless a limitation of our approach, as no empirical studies exist validating the use of this probability distribution in ALE meta-analyses for LSM data.

## RESULTS

### ALE Meta-analysis of Global Accuracy

Descriptions of the 10 papers used for ALE meta-analysis studying accuracy in naming are shown in Table 1. These papers in total regarded 69 foci, acquired from 534 subjects. As far as we could establish, within each paper coverage over left temporal and left inferior frontal areas was similar; as such, there was in general no particular bias to frontal cortex relative to temporal cortex.

**Table 1.** List of papers included for anatomic likelihood analysis meta-analysis, studying global accuracy in naming.

Study	*N* (#f)^c^	Age (range or *M* ± *SD*)	Post-stroke time (months)	Language	Dependent variable	Lesion delineation	Modality	Analysis	Lesion volume as covariate	Min. subjects with lesioned voxel^d^	No. of foci (and cluster contribution)^e^	Quality (/13)
Akinina et al., 2019	40 (21)	33–78	>3	Russian	Verb naming accuracy (picture)	Manual	MRI	VLSM	Yes	10% (*n* = 4)	1 (1)	12
Alyahya et al., 2018b	48 (14)	44–87	>12	English	Noun naming accuracy (picture)	Automated	MRI	VBCM	Yes	n.m.	8 (1, 2)	12
Baldo et al., 2013	96 (21)	31–84	>3	English	Noun naming accuracy (picture)	Manual	MRI, CT	VLSM	n.m.	5% (*n* = 5)	1 (2)	12
Faroqi-Shah et al., 2014	31 (10)	42–72	>10	English	Noun naming accuracy (picture)	Manual	MRI	VLSM	n.m.	13% (*n* = 4)	5 (2, 3)	11
Geva et al., 2012 ^a^	21 (7)	21–81	>6	English	Noun naming accuracy (CAT)	Manual	MRI	VLSM	Yes	5% (*n* = 1)	12* (2, 3, 4)	12
Griffis et al., 2017	43 (18)	23–90	>12	English	Noun naming accuracy (picture)	Automated	MRI	SVR-LSM	Yes	23% (*n* = 10)	13 (1, 2, 3)	12
Lukic et al., 2021	76 (26)	22–81	>8	English	Verb naming accuracy (NNB)	Semiautomated	MRI	VLSM	Yes	10% (*n* = 7)	2 (1, 3)	13
Piras & Marangolo, 2010	20 (7)	38–78	>6	Italian	Noun naming accuracy (picture)	Manual	MRI	VLSM	Yes	25% (*n* = 5)	5 (1, 3)	13
Sul et al., 2019	31 (15)	55.5 ± 11.5	>12	Korean	Naming score (K-WAB)	Manual	MRI	VLSM	n.m.	10% (*n* = 3)	7 (1, 2)	9
Thye & Mirman, 2018 ^b^	128 (57)	26–79	>1	English	Noun naming accuracy (PNT)	Manual	MRI, CT	VLSM	Yes	10% (*n* = 13)	15* (1, 3, 4)	10

*Note*. Post-stroke time was the minimum time between stroke onset and scanning or testing, whichever was performed earlier. Quality assessment was performed by scoring different parameters out of a maximum of 13 points (details and weighted distributions of points can be found in Table S1, scoring per paper can be found in Table S2). CAT = Comprehensive Aphasia Test; NNB = Northwestern Naming Battery; K-WAB = Korean version of the Western Aphasia Battery; PNT = Philadelphia Naming Test; MRI = magnetic resonance imaging; CT = computed tomography; VLSM = voxel-based lesion-symptom mapping; VBCM = voxel-based correlational methodology; SVR-LSM = support vector regression multivariate lesion-symptom mapping; n.m. = not mentioned in paper or supplementary material.

^a^ For Geva et al. (2012), the statistical map was cluster thresholded at z > 4.61 by the first author of that study.

^b^ For Thye and Mirman (2018), from the statistical map made available, center coordinates of clusters with >10 voxels were selected by the authors, in a procedure blinded for cluster/voxel location.

^c^ Number of subjects with the amount of females stated in brackets.

^d^ Minimum number of subjects with lesion in a specific voxel before this voxel is included in statistical analysis, presented as percentage out of the full cohort.

^e^ Number of significant foci obtained.

Quality of the evidence was assessed and total score per paper can be found in Table 1. Papers scored 9 or higher. Detailed scoring per quality parameter can be found in Table S2.

**Figure 2.**
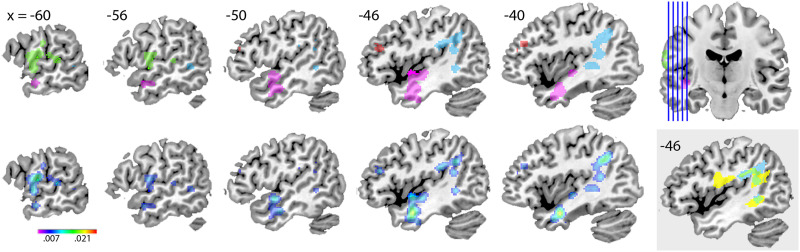
Results of the anatomic likelihood estimate (ALE) analysis. (Top) Location of the four clusters (cluster 1 in pink, cluster 2 in light blue, cluster 3 in green, cluster 4 in red), with significant association between global accuracy in naming task performance and brain lesions. Location of all sagittal slices is indicated in the upper right corner. (Bottom) ALE maps of the four clusters, corrected for cluster-level familywise error at an alpha level of 0.05 (following voxel-level threshold of 0.01). The color bar indicates the ALE value range. (Gray inset) Cluster 2 (in blue), arcuate fasciculus (in yellow), and their overlap (in green). The arcuate fasciculus mask was obtained from the Natbrain atlas (Catani & Thiebaut de Schotten, 2008).

Results of the ALE analysis can be found in Figure 2 and Table 2. Here, we follow the subdivision of the temporal lobe into anterior, mid, and posterior portions by Indefrey and Levelt (2004), with corresponding boundary *y* coordinates in Talairach space at −7 and −38. Four significant clusters were identified. Cluster 1, with 7 peaks contributed by seven studies, had the maximal ALE value (ALE = 0.028) in left anterior temporal cortex (MNI −42, −2, −24), closest to MTG. Cluster 2, with 10 peaks contributed by six studies, had the maximal ALE value (ALE = 0.016) in left inferior parietal lobule (labeled angular gyrus in the AAL atlas; Devenyi et al., 2017). Cluster 3, with 5 peaks contributed by six studies, had the maximal ALE value (ALE = 0.017) in left postcentral gyrus. Cluster 4, with 5 peaks contributed by two studies, had the maximal ALE value (ALE = 0.011) in left.

**Table 2.** Significant anatomic likelihood analysis clusters and corresponding MNI coordinates of the local maxima.

Cluster	Volume (mm^3^)	Label	ALE score	*Z* score	Coordinates		
					*x*	*y*	*z*
1	6,736	Left Superior Temporal Gyrus, BA 38	0.028	6.3	−42	−2	−24
		Left Superior Temporal Gyrus, BA 22	0.013	3.9	−48	−2	−8
		Left Sub-lobar Insula, BA 13	0.012	3.8	−44	−14	−6
		Left Inferior Temporal Gyrus, BA 20	0.010	3.5	−50	−10	−22
		Left Inferior Temporal Gyrus, BA 20	0.010	3.5	−44	−8	−34
		Left Inferior Temporal Gyrus, BA 21	0.010	3.4	−60	−8	−16
		Left Middle Temporal Gyrus, BA 21	0.008	2.9	−54	2	−16
2	6,208	Left Superior Temporal Gyrus, BA 39	0.016	4.5	−42	−48	32
		Arcuate Fasciculus*	0.011	3.6	−40	−46	14
		Arcuate Fasciculus*	0.011	3.6	−46	−48	0
		Left Sub-lobar Insula, BA 13	0.011	3.6	−46	−30	24
		Left Caudate Tail	0.010	3.6	−38	−40	2
		Left Sub-lobar Insula, BA 13	0.010	3.5	−44	−36	28
		Left Sub-lobar Insula, BA 13	0.010	3.3	−38	−40	26
		Left Transverse Temporal Gyrus, BA 41	0.009	3.2	−36	−38	12
		Left Caudate Tail	0.009	3.2	−38	−32	2
		Left Middle Temporal Gyrus, BA 22	0.008	2.9	−56	−48	2
3	3,816	Left Transverse Temporal Gyrus, BA 42	0.017	4.5	−62	−10	14
		Left Superior Temporal Gyrus, BA 22	0.012	3.8	−62	−6	0
		Left Postcentral Gyrus, BA 2	0.010	3.4	−62	−16	26
		Left Superior Temporal Gyrus, BA 42	0.010	3.4	−60	−30	8
		Left Postcentral Gyrus, BA 40	0.008	2.9	−62	−24	14
4	2,360	Left Middle Frontal Gyrus, BA 9	0.011	3.7	−30	40	20
		Left Middle Frontal Gyrus, BA 9	0.010	3.5	−34	36	26
		Left Middle Frontal Gyrus, BA 10	0.010	3.5	−36	44	18
		Left Middle Frontal Gyrus, BA 46	0.010	3.5	−46	34	20
		Left Middle Frontal Gyrus, BA 9	0.010	3.5	−42	36	26

*Note*. BA = Brodmann Area. Anatomical labeling provided by BrainMap GingerALE 3.0, based on the Talairach Daemon 1988 atlas. MNI = Montreal Neurological Institute.

* No gray matter found, anatomical label derived from the Natbrain atlas (Catani & Thiebaut de Schotten, 2008).

### Beyond-Accuracy Analysis

Details of the five papers considered for beyond-accuracy analysis can be found in Table 3. These papers measured different dependent variables. We tried to link their word production measure to proposed stages of word production (Dell, 1986; Levelt et al., 1999; Schwartz et al., 2004). We note that on the basis of the outcome measures reported, conceptual preparation and lexical selection could not be distinguished.

**Table 3.** List of papers studying a beyond-accuracy measure of naming.

Study	*N* (#f)^a^	Post-stroke time (months)	Language	Dependent variable	Stage of word production
Dell et al., 2013	103 (44)	>1	English	Semantic and phonological parameter weights via computational modelling	Conceptual/lexical selection and phonological encoding respectively
Fridriksson et al., 2018	105 (n.m.)	>6	English	Semantic and phonological noun naming errors	Conceptual/lexical selection and phonological encoding respectively
Harvey & Schnur, 2015	15 (4)	>6	English	Semantic interference in noun naming	Conceptual/lexical selection
Schnur et al., 2009	12 (n.m.)	>10	English	Growth of semantic interference in noun naming	Conceptual/lexical selection
Tochadse et al., 2018	53 (n.m.)	>12	English	Semantic and phonological parameter weights via computational modelling	Conceptual/lexical selection and phonological encoding respectively

*Note*. Post-stroke time was the minimum time between stroke onset and scanning or testing, whichever was performed earlier. n.m. = not mentioned in paper or supporting information.

^a^ Number of subjects with the number of females stated in brackets.

Out of the papers included for beyond-accuracy analysis, five papers studied a measure most likely associated with the conceptual-lexical selection stage, and three papers also studied a measure most likely associated with the phonological encoding stage.

Fridriksson et al. (2018) studied semantic and phonological errors, linked to the conceptual-lexical and phonological code stages, respectively. Lesion-symptom mapping results for semantic errors revealed significant areas overlapping with cluster 2, the strongest predictor of semantic error production being lesions in the left “posterior” (authors’ own terminology) MTG. No significant regions were found to be predictive of phonological errors made in naming (cf. Schwartz et al., 2012). Of note, our cluster 3 shows overlap with their LSM results for articulation rate.

A different way to examine lexical-semantic versus phonological stages is through formalization of a computational model. Based on Dell’s computational model of word production (Dell, 1986; Dell et al., 2013), two relevant parameters are defined: (1) The *s* parameter, representing the connection weights between conceptual and lexical units (“lemma access”), and (2) the *p* parameter, the connection weights between the lexical and phonological units. From performance in picture naming and nonword repetition tests, Dell et al. (2013) derived *s* and *p* parameters, which were analyzed with VLSM. The *s* parameter was associated with left “anterior” (authors’ own terminology) STG and MTG, left temporal pole, and left middle and inferior frontal gyri, overlapping with our four clusters. In addition, the association was also present posteriorly, at the temporo-parietal and parietal-temporal-occipital junctions, including AG. The *p* parameter was mainly associated with left SMG and postcentral gyrus (also including precentral gyrus and insula, similar to our cluster 3). Tochadse et al. (2018) similarly studied naming through *s* and *p* weights from Dell’s computational model. Based on the peak coordinates obtained in their VBCM analysis and coordinates for the subdivision of the temporal lobe (based on Indefrey & Levelt, 2004), the *s* parameter was associated with regions in the mid portion of the left temporal lobe. Peak coordinates associated with the *p* parameter were located either in the anterior or mid portions of the temporal lobe.

Two other papers combined LSM with semantic interference, which could be linked to the conceptual-lexical selection stages of word production (Roelofs, 2018). Harvey and Schnur (2015) studied the areas involved in both semantic interference and growth of interference across cycles in naming. The largest significant cluster associated with semantic interference was located in the left posterior MTG (according to the subdivision adopted here: MNI −52, −40, −5, Talairach *y* = −40), close to our cluster 2, whilst the other cluster was located in the left mid MTG (according to the subdivision adopted here: MNI −49, −21, −8, Talairach *y* = −22). No region was significantly associated with growth in interference across naming cycles. This latter dependent variable, that is, the growth of interference, was specifically studied in a study by Schnur et al. (2009). VLSM analysis revealed that growth of interference was significantly related to voxels only in the “posterior” (author’s own terminology) left IFG.

To conclude the beyond-accuracy analysis, a tendency seems to be present across studies for deficits in the conceptual preparation and/or lexical retrieval stages to be associated with lesions in somewhat more mid to posterior temporal regions. Regarding the phonological code retrieval stage, since only two studies obtained statistically significant results (Dell et al., 2013; Tochadse et al., 2018) that were not converging, the evidence remains inconclusive.

## DISCUSSION

To investigate the brain areas critical for word production, the present study quantitatively compared results from papers combining lesion-symptom mapping with global accuracy scores in naming, by performing an ALE meta-analysis. We identified four separate clusters. One cluster was predominantly in the anterior portion of the left temporal lobe, in STG, MTG, and ITG. The second cluster was predominantly in the posterior portion of the left temporal lobe including the inferior parietal lobule, mostly in white matter. An overlay of this cluster with the outline of the arcuate fasciculus indicated a large degree of overlap. The third cluster had a peak in postcentral gyrus and the fourth cluster in middle frontal gyrus. No peaks were identified in the left IFG. This distribution was found despite a similar lesion coverage over left inferior frontal as over left temporal lobe areas. In general, the quality of the evidence across studies was good for the purpose of our review and meta-analysis. The vast majority of the studies were conducted in English-speaking countries, with only three other languages represented in our sample.

Three papers could not be included in the global accuracy due to missing coordinates. The three strongest predictors of correct naming obtained by Fridriksson et al. (2018) were the left “posterior” (authors’ own terminology) STG, AG, and SMG. Anterior portions of the temporal lobe were also found in this study, though these predictors were less strong. Pillay et al. (2017) provided a VLSM map of picture naming in their supplementary materials, which revealed significant areas (through visual inspection) in posterior portions of left STG, inferior parietal lobule, lateral frontal cortex, and insula. Finally, in the study by Skipper-Kallal et al. (2017), impairment in picture naming was associated with left posterior STG (authors’ own terminology) in particular, but also with left AG, intraparietal sulcus, and parts of the pars triangularis in the IFG.

Since overall naming scores reflect a mixture of errors, it is difficult to relate the patterns found to particular stages of word production. For example, while Akinina et al. (2019) took care to try to isolate the lexical stage, and Baldo et al. (2013) reported their findings while covarying for visual perception and overall speech fluency deficits, the global accuracy measure in other studies is less specific to one or a couple of stages. Therefore, we also synthesized studies examining measures beyond global accuracy in an attempt to elucidate the patterns found by relating them as much as possible to particular stages of word production as stipulated by psycholinguistic models. We found tentative evidence that conceptual preparation and/or lexical selection are associated with lesions in somewhat more mid to posterior temporal lobe regions, whereas the evidence for phonological encoding was less consistent across studies.

In the course of publishing this work, another meta-analysis of lesion-symptom mapping studies was published focusing on various language tasks (Na et al., 2022). For naming, the authors found a cluster in the left parahippocampal gyrus and left mid STG (MNI −59, −11, 7, Brodman Area 22, Talairach *y* = −12). This cluster is in the proximity of cluster 3 we identified. However, unlike in our meta-analysis, the authors did not differentiate between phase of the stroke (acute, subacute, and chronic were all included) or performance measure (global accuracy as well as specific error types were included) in the analysis.

While two previous (semi-)systematic reviews and meta-analyses have provided evidence on the neural substrates of more specific stages of word production based on correlational measures (Indefrey & Levelt, 2004; Price, 2012), here we explicitly sought to provide causal evidence. The meta-analysis of Indefrey and Levelt (2004) has suggested that lexical selection is associated with left MTG (and the mid portion in particular), whereas phonological code retrieval (part of phonological encoding) is associated with left posterior MTG and STG. Syllabification (the ordering of phonemes into syllables, part of phonological encoding) is associated with left posterior IFG and phonetic encoding and articulation mainly with bilateral ventral motor and sensory regions. Our results for global naming accuracy are partly in line with this proposal. The beyond-accuracy analysis, by contrast, provides tentative evidence in agreement with this proposal regarding the conceptual/lexical stages, which in our findings were related to more mid to posterior temporal lobe regions.

Our results concern stroke populations only. However, given that different pathologies have their intrinsic spatial biases—in the case of stroke, due to cerebrovascular organization—trying to bridge across pathologies would be fruitful for our understanding of the neural basis of language production. These studies are however scarcer, so no meta-analysis is possible given the current literature. Of note, converging evidence for the present results can be found. For example, in an LSM study examining presurgical brain tumor cases (Faulkner & Wilshire, 2020), conceptual/lexical selection (operationalized as semantic errors and omissions in picture naming and category fluency performance covaried for letter fluency performance) was associated with left posterior MTG and parts of the lateral occipital cortex and ITG. Phonological encoding (operationalized as accuracy on word and nonword repetition and effect of word length in picture naming) was associated with left posterior SMG and AG. In individuals with Alzheimer disease, correlations between hypometabolism and naming errors have also been found (Isella et al., 2020). In particular, semantic errors were related to the mid portion of the MTG and left ITG. Formal errors (i.e., the resulting existing word resembles the target word in terms of its form, “rat” instead of “mat”), tapping both into the connections between lexical and phonological units and into phonological encoding (Dell et al., 1997), were associated with left anterior/mid MTG. Finally, producing neologisms and nonwords was associated with left SMG and the mid portion of STG. In a cohort of individuals with primary progressive aphasia producing semispontaneous speech (Wilson et al., 2010), atrophy in the mid portion of the left temporal lobe (mainly MTG) was associated with the production of nouns of increasing lexical frequency, a measure tapping into both lexical and phonological stages (Kittredge et al., 2008). In turn, producing phonological paraphasias was associated with atrophy in the mid portion of left STG. In sum, converging with the stroke-aphasia literature reviewed above, conceptual/lexical stages tend to be associated with the mid to posterior portions of the temporal lobe, and MTG in particular, in addition to ITG. This latter region is not often represented in stroke-aphasia cases given the difference in arterial blood supply between STG and MTG on the one hand, and ITG on the other. Once phonological representations become implicated, a tendency is seen for associations with (mid) STG and SMG, which is also suggested by some of the stroke studies we reviewed.

Overall, the literature strongly suggests an important role for the left temporal, rather than frontal, lobe in naming, contrary to a perhaps more popular view of the left temporal lobe as the site for comprehension and the frontal lobe for production. Part of this misconception may have its roots in the fact that producing language is a *motor* function, which nevertheless requires the retrieval of conceptual, lexical, and phonological information, processes that we would argue are not particularly linked to the frontal cortex.

Admittedly, the vast majority of the studies reviewed here employed noun rather than verb naming (with the exception of Akinina et al., 2019; Lukic et al., 2021), a deliberate choice to increase the comparability across papers. A review of a large body of literature comparing nouns and verbs has concluded that these two grammatical classes are processed by a largely overlapping set of areas in production and comprehension (Vigliocco et al., 2011). Neural differences between the two emerge, however, as a function of the task (among a few other variables, see Vigliocco et al., 2011): for example, when there is an emphasis in morphosyntax as in verb naming, in which case the involvement of left IFG becomes more prominent. In both Akinina et al. (2019) and Lukic et al. (2021), the accuracy score was based on any correct morphological form of the verb, thus emphasizing more morphosyntax than noun/object naming does. Thus, one could argue that our inclusion of more studies examining noun naming rather than verb naming may have overemphasized the temporal rather than the frontal cortex. However, the frontal cortex contribution in this case would arguably not be due to conceptual-lexical (and phonological) information being retrieved. Thus, our conclusion remains that the left temporal, rather than the frontal cortex, is the most critical for conceptually driven naming.

The terminology adopted by authors for subdividing the temporal lobe is not always consistent or transparent. Here, we opted for adopting a coordinate-based standard used by Indefrey and Levelt (2004), separating the temporal lobe into an anterior, a mid, and a posterior portion. This system is useful for being more objective (when coordinates are available). However, the boundaries between these portions are not necessarily a subdivision reflecting some form of organization of the temporal lobe (a “natural kind,” cf. cytoarchitectonics, transcriptomics, etc.), but rather a convention adopted to help researchers structure results. Future research could attempt to link findings of particular locations in the temporal lobe to physiologically and biologically based subdivisions, which could prove useful for integrating findings across studies using different methods and elucidating temporal lobe functions.

A limitation of our study is the small number of comparable papers in the analyses performed. We used strict criteria whereby papers were excluded if there was cohort overlap or if we could not ascertain that there was no overlap. Hence, more empirical studies are required to increase robustness of the ALE meta-analysis and to strengthen our claims on the psychological nature of the foci identified in the present study. These limitations relate to two recommendations we can make to improve the field of language production. Firstly, authors would ideally disclose cohort overlap with previous studies (this was done in some, but not all, of the studies we reviewed). Secondly, following open science practices, authors would ideally make postprocessed data available (e.g., Thye & Mirman, 2022). An alternative to this solution would be to report a set of coordinates for as much as the method affords. A second limitation of our study is the use of the ALE method without a validation for the probability distribution we employed in combination with LSM data.

In conclusion, the ALE meta-analysis of 10 lesion-symptom mapping studies of naming performance yielded distinct clusters, predominantly in anterior and posterior portions of the left temporal lobe, for which the posterior distribution seems to follow the arcuate fasciculus. Two additional clusters were found in postcentral and middle frontal gyrus. No peaks were identified in the left IFG. Regions consistent with these foci were also revealed by examining papers studying more detailed measures of naming or other populations than stroke, where we found a tendency for lesions in mid to posterior parts of the temporal lobe to be more consistently associated with conceptual-lexical deficits. A major limitation of the present study remains the small number of papers included in the meta-analysis.

## ACKNOWLEDGMENTS

The authors are indebted to Peter Indefrey for critical discussions, Sharon Geva, Daniel Mirman, Melissa Thye, Dorian Pustina, Sladjana Lukic, and Laura Skipper-Kallal for providing additional information for the meta-analysis, and Daniel Sharoh for a blinded procedure of cluster threshold of one data set. The authors are also thankful to the critique provided by three anonymous reviewers, which substantially improved the quality of the work presented here.

## FUNDING INFORMATION

Vitória Piai, Dutch Research Council, Award ID: NWO, 451-17-003. Vitória Piai, Language in Interaction Consortium, Dutch Research Council, Award ID: NWO, 024.001.006.

## AUTHOR CONTRIBUTIONS

**Vitória Piai**: Conceptualization; Formal analysis; Project administration; Visualization; Writing – original draft; Writing – review & editing. **Dilys Eikelboom**: Formal analysis; Project administration; Visualization; Writing – original draft; Writing – review & editing.

## DATA AVAILABILITY STATEMENT

All data associated with these analyses are available via https://osf.io/8xtp9/.
