# Supplementary material for: Brain Areas Critical for Picture Naming: A Systematic Review and Meta-Analysis of Lesion-Symptom Mapping Studies
Source: Neurobiol Lang (Camb). 2023 Apr 11;4(2):280–96. doi: 10.1162/nol_a_00097 (PMC10205157; doi:10.1162/nol_a_00097)
Supplement: Supplementary file 1 [file nol-4-2-280-s001.docx]

**Supplementary material**

***Table S1. Quality assessment - Detailed terms and weighted distribution of points.***

| **1. Was the study population clearly specified and defined?** | **/6** |
| --- | --- |
| - Is it clearly a stroke cohort? | /1 |
| - Is it clearly a single-stroke cohort? | /1 |
| - Is there a clear description of the timing since stroke? | /1 |
| - Is there a clear description of the handedness of the participants? | /1 |
| - Is there a clear description of the lesion locations?   - Is it clear in which hemisphere the lesions reside?   - Is there a lesion overlap map present? | /1  /1 |
| **2. Were the exposure measures clearly defined, valid, reliable and implemented consistently across all study participants?** | **/2** |
| - Is there a clear description of the used naming task? | /1 |
| - Is there a clear description of how performance is scored? | /1 |
| **3. Is there a clear description of the statistical analysis?** | **/4** |
| - Is there a clear description of the lesion-symptom mapping method used? | /1 |
| - Is lesion volume used as a covariate? | /1 |
| - Are other confounding variables implemented as covariates? | /1 |
| - Is there a clear description of how the researchers accounted for multiple comparisons? | /1 |
| **4. Is the outcome measure* clearly specified?** (*lesioned brain areas correlated with deficits in naming performance) | **/1** |
| **Total** | **/13** |

| **Table S2.** Quality assessment – Scoring per paper. | | | | | | | | | | | | | |
| --- | --- | --- | --- | --- | --- | --- | --- | --- | --- | --- | --- | --- | --- |
| **Study** | **Population** |  |  |  | **Lesion location** |  | **Exposure measures** |  | **Statistical analysis** |  |  |  | **Outcome measure** |
|  | Stroke | Single stroke | Timing since stroke | Handedness | Which hemisphere | Lesion overlap map | Naming task description | Performance scoring | LSM method | Lesion volume as covariate | Other covariates | Multiple comparison correction |  |
| Akinina et al., 2019 | * |  | * | * | * | * | * | * | * | * | * | * | * |
| Alyahya et al., 2018a | * | * | * | * | * | * | * | * | * | * |  | * | * |
| Baldo et al., 2013 | * | * | * | * | * | * | * | * | * |  | * | * | * |
| Faroqi-Shah et al., 2014 | * | * | * | * | * | * | * | * | * |  | * |  | * |
| Geva et al., 2012 | * |  | * | * | * | * | * | * | * | * | * | * | * |
| Griffis et al., 2017 | * | * | * | * | * | * | * | * | * | * |  | * | * |
| Lukic et al., 2021 | * | * | * | * | * | * | * | * | * | * | * | * | * |
| Piras & Marangolo, 2010 | * | * | * | * | * | * | * | * | * | * | * | * | * |
| Sul et al., 2019 | * | * | * | * | * | * |  |  | * |  |  | * | * |
| Thye & Mirman, 2018 | * | * | * | * | * | * |  |  | * | * |  | * | * |

*Note.* LSM = lesion-symptom mapping. For detailed terms see Table S1 of the supplementary material.
